# Association between uterine artery Doppler velocimetry, placenta accreta spectrum, and depth of placental implantation

**DOI:** 10.1002/pmf2.70436

**Published:** 2026-08-27

**Authors:** Elias Kassir, Edgar A. Hernandez‐Andrade, Donatella Gerulewicz‐Vannini, Neha Agarwal, Sarah T. Mehl, Eleazar E. Soto‐Torres, Ramesha Papanna, Sean C. Blackwell, Baha M. Sibai, Farah H. Amro

**Affiliations:** ^1^ Department of Obstetrics Gynecology and Reproductive Sciences McGovern Medical School University of Texas Health Science Center at Houston (UTHealth) Houston Texas USA

**Keywords:** color Doppler, hysterectomy, lower uterine segment, obstetrics, placenta accreta spectrum, ultrasound, uterine artery

## Abstract

**Introduction:**

We aim to evaluate uterine artery (UtA) Doppler velocimetry in patients at risk of placenta accreta spectrum (PAS).

**Methods:**

Prospective cohort of patients referred to an academic referral center for suspected PAS. The left and right UtA were evaluated, and mean peak systolic velocity (PSV), end‐diastolic velocity (EDV), and pulsatility index (PI) were obtained and converted to gestational age (GA)‐adjusted *z*‐scores. Differences in UtA Doppler indices between patients with and without PAS, and among varying degrees of placental implantation depth, were analyzed. The predictive performance of UtA Doppler velocimetry to diagnose PAS and intraoperative bleeding (IOBL) >1500 mL was calculated. A sub‐analysis of patients with and without placenta previa was conducted.

**Results:**

A total of 177 patients were included, and PAS was confirmed in 61.0% (108/177). In the complete study group, patients with PAS had significantly lower UtA‐PI and higher UtA‐PSV and UtA‐EDV (*p* < 0.05) than patients without PAS. We found the following associations with PAS: UtA‐PSV ≥90th percentile for GA demonstrated an odds ratio (OR) of 4.9 (95% confidence interval [CI], 2.31–10.39; *p* < 0.0001); UtA‐EDV ≥90th percentile for GA demonstrated an OR of 3.49 (95% CI, 1.71–7.14; *p* = 0.0006); and UtA‐PI ≤25th percentile for GA demonstrated an OR of 0.71 (95% CI, 0.23–2.23; *p* = 0.2). The predictive performance of UtA velocities improved when analyzing only patients without placenta previa. There were no significant differences in UtA Doppler indices related to the placental implantation depth.

Patients with severe IOBL (>1500 mL, *n* = 86) had significantly higher UtA‐PSV (*p* = 0.004) and UtA‐EDV (*p* = 0.0001), but no differences in UtA‐PI than those without severe IOBL (*n* = 79). The association between UtA‐PSV ≥90th percentile for GA and severe IOBL demonstrated an OR of 3.67 (95% CI, 1.86–7.23; *p* = 0.002); UtA‐EDV ≥90th percentile for GA demonstrated an OR of 3.34 (95% CI, 1.68–6.64; *p* = 0.0006). The association between increased UtA velocities and severe IOBL was similar regardless of the presence of placenta previa.

**Conclusions:**

Patients with PAS showed lower UtA‐PI and higher UtA‐PSV and UtA‐EDV than those without PAS. The predictive performance of UtA Doppler velocimetry for PAS seems to improve in patients without placenta previa. Patients who experienced severe IOBL exhibited significantly higher UtA‐PSV and UtA‐EDV than those without severe IOBL.

## 1 INTRODUCTION

Placenta accreta spectrum (PAS) is associated with substantial maternal morbidity, primarily due to severe obstetric hemorrhage [1, 2, 3, 4, 5]. Two major factors contributing to increased peripartum complications in PAS are failure to establish the diagnosis antenatally and greater depth of placental invasion (e.g., placenta percreta) [6, 7].

Clinical suspicion of PAS is mainly based on two key factors: prior cesarean delivery and the presence of placenta previa [8]. The risk increases proportionally with the number of previous cesarean deliveries [9]. Additional risk factors include prior uterine surgery, recurrent miscarriages, uterine curettage, and pregnancies conceived through in vitro fertilization (IVF) [10, 11]. Patients presenting with these risk factors should undergo detailed placental imaging to assess for sonographic signs of PAS.

Many ultrasound findings associated with PAS reflect abnormalities in uteroplacental vascularity [12]. Classic signs such as placental lacunae are thought to result from high‐velocity blood flow entering the chorionic tissue [13]. Furthermore, in patients with suspected PAS, increasing uteroplacental hypervascularity has been associated with deeper placental invasion and greater intraoperative blood loss (IOBL) at the time of delivery [14]. Color Doppler imaging is the standard modality used to evaluate placental and uterine vascularization.

The uterine arteries (UtAs) provide the primary blood supply to the gravid uterus. Combined UtA blood flow increases from approximately 20–30 mL/min in the nonpregnant state to 600–900 mL/min at term [15, 16]. Being the main source of blood flow to the uterus and placenta, it is plausible that UtA hemodynamics may be altered in the presence of PAS. However, data evaluating UtA Doppler velocimetry in PAS are limited [17, 18, 19, 20].

In this study, we aim to evaluate differences in UtA Doppler indices between patients with and without PAS, and to determine whether these parameters are associated with PAS severity and IOBL at the time of cesarean hysterectomy.

## 2 METHODS

### 2.1 Study design

This prospective cohort study included consecutive patients referred to a single academic quaternary referral center for suspected PAS between December 2021 and June 2025. The study was approved by the Institutional Review Board of the University of Texas Health Science Center at Houston (HSC‐MS‐18‐0968).

All referred patients underwent comprehensive ultrasound evaluation at our center. We included patients who were evaluated for suspected PAS and subsequently received prenatal care and delivered at our institution, regardless of whether they were classified as low‐ or high‐risk for PAS after the initial assessment. We excluded patients who continued care and delivered elsewhere, as well as those managed conservatively (i.e., placenta intentionally left in situ following cesarean delivery) [21, 22].

### 2.2 Ultrasound examinations

Ultrasound examinations were performed using GE Voluson E10 and Samsung Hera W10 systems equipped with 1–6 MHz curved‐array transabdominal and 2–5 MHz transvaginal probes. Complete fetal anatomical survey and biometry were obtained. The placenta and the lower uterine segment were evaluated using transabdominal and transvaginal ultrasound with the aid of color Doppler. The entire placenta was systematically scanned in lateral and craniocaudal sweeps. The uteroplacental interface and placental parenchyma were carefully assessed [23]. Patients were considered at risk of PAS when one or more of the following sonographic signs were present: loss of the retroplacental hypoechoic zone, placental lacunae, placental bulging, placental thickening, bridging vessels, abnormalities of the uterine serosa–bladder interface, or increased vascularity [12].

### 2.3 UtA Doppler assessment

**FIGURE 1:**
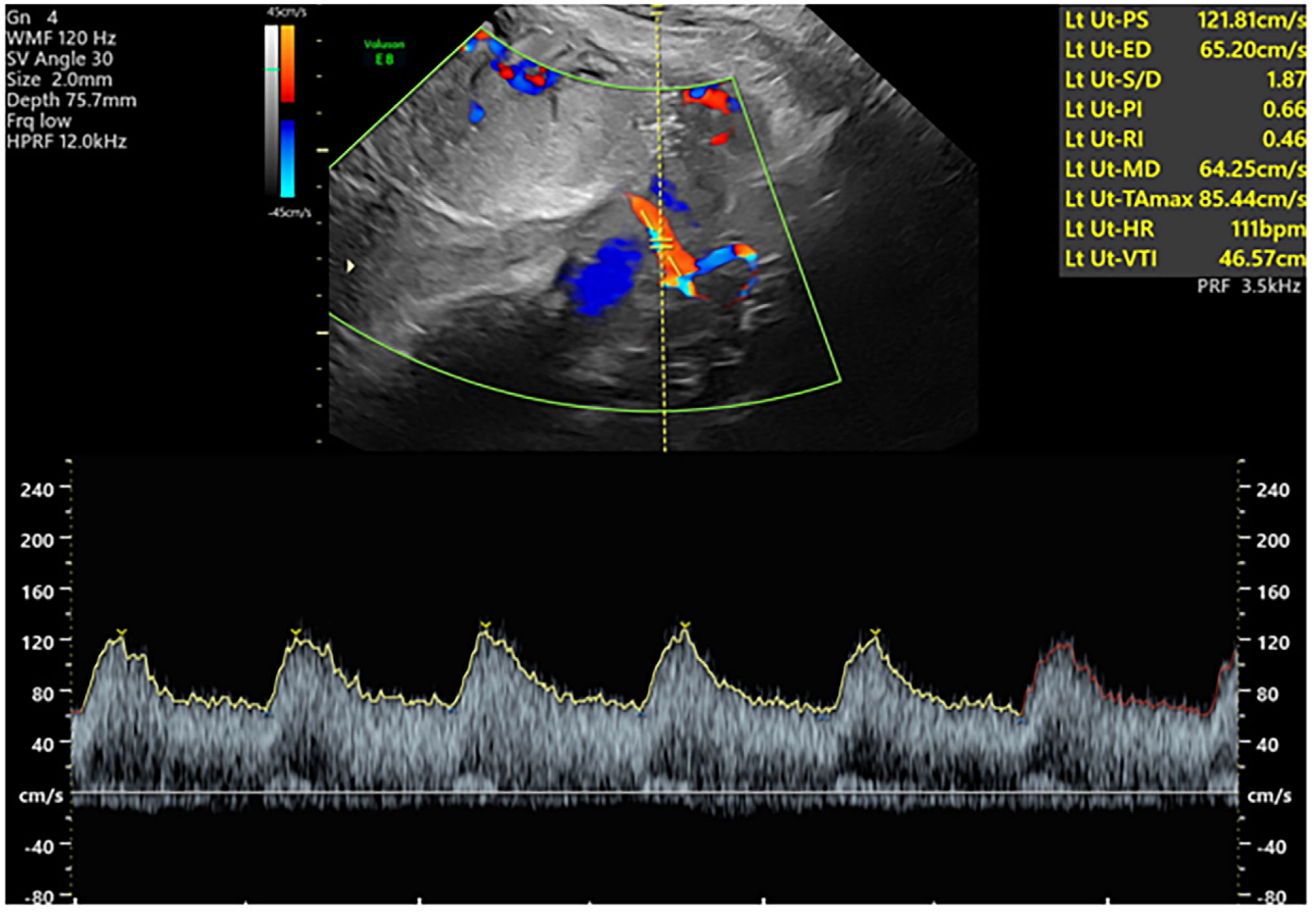
Sample image of the interrogation of the right uterine artery.

The UtAs were assessed transabdominally in a parasagittal plane lateral to the uterus. Using color directional Doppler, the UtAs were identified at their apparent crossover with the external iliac artery. The pulsed‐wave Doppler sample volume was placed in the mid‐portion of the vessel approximately 1 cm distal to this crossing point. A wall filter of 90 Hz was applied. The angle of insonation was maintained as close to 0° as possible and angle‐corrected when necessary, but never greater than 45°, due to the trajectory of the UtA. Pulse repetition frequency was adjusted so that approximately 75% of the Doppler display was occupied by the velocity waveform (Figure 1). Three to five consecutive uniform waveforms were obtained. The following parameters were recorded bilaterally: pulsatility index (PI), peak systolic velocity (PSV), and end‐diastolic velocity (EDV). Mean values were calculated by averaging left and right measurements [18, 24].

Determination of PAS suspicion was made by board‐certified maternal‐fetal medicine specialists based on ultrasound findings and clinical risk factors (e.g., prior cesarean delivery, prior uterine surgery). While the clinicians were not blinded to the images of UtA Doppler interrogation, the indices were not used for clinical risk stratification.

### 2.4 Variables and outcomes

Demographic, ultrasound, intraoperative, and pathologic data were collected via structured chart review and entered into REDCap [25]. For analysis, only Doppler data from the initial ultrasound performed at our center were used. Collected variables included: maternal demographics, the presence of placenta previa, UtA Doppler indices, PAS and risk classification (low vs. high), IOBL, histopathologic confirmation of PAS, and depth of placental invasion (accreta vs. increta vs. percreta).

The primary outcome was histopathologic confirmation of PAS following delivery. Secondary outcomes included: depth of placental invasion and severe IOBL (>1500 mL). PAS diagnosis and severity were confirmed by histopathologic examination following hysterectomy. Patients in whom the placenta separated spontaneously without need for hysterectomy were classified as not having PAS. IOBL was determined using a combination of visual estimation of blood loss, blood in the suction canister, and blood on laparotomy sponges weighed out by nursing staff to arrive at a quantitative blood loss. Patients for whom there were conflicting numbers for IOBL in the intraoperative documentation, in all cases due to inconsistencies in the estimated vaginal blood loss prior to arrival in the operating room, were excluded from any analyses involving IOBL.

### 2.5 Statistical analysis

Analyses were performed for the overall cohort and stratified by placenta previa status. Descriptive statistics were used to report demographic characteristics of the study groups. UtA Doppler indices were converted to gestational age–adjusted *z*‐scores using established reference values. Reference ranges for UtA‐PSV and UtA‐EDV were derived from a previously analyzed cohort of 500 uncomplicated pregnancies (Table S1). We used previously published reference ranges for UtA‐PI values for normal pregnancies [26].

Comparisons of Doppler parameters between patients with and without PAS, among patients with different PAS severity, and between patients with and without severe IOBL (>1500 mL) were performed. Continuous variables were compared using Student's *t*‐test or ANOVA with post hoc analysis as appropriate. Receiver operating characteristic (ROC) curve analysis was performed to evaluate the predictive performance of individual Doppler parameters for PAS and severe IOBL. Associations between UtA Doppler indices and PAS and severe IOBL were assessed using logistic regression, with results reported as odds ratios (ORs) and 95% confidence intervals (CIs). Statistical analyses were conducted using SPSS (IBM Corp.) and MedCalc (MedCalc Software Ltd.). A two‐sided *p* value < 0.05 was considered statistically significant.

## 3 RESULTS

Demographic characteristics are presented in Table 1. A total of 177 patients were included. Placenta previa was present in 129/177 (72.9%) and absent in 48/177 (27.1%). Maternal characteristics did not differ significantly between those with and without placenta previa. PAS was confirmed in 108/177 cases (61.0%): 14 accreta, 66 increta, and 28 percreta. The proportion of severe PAS (increta/percreta) was higher than that of accreta, likely reflecting referral bias to a quaternary center and/or differences in pathologic classification. PAS was significantly more frequent among patients with placenta previa compared with those without previa (98/129 [76.0%] vs. 10/48 [20.8%]; *p* < 0.0001).

**TABLE 1:** Characteristics of the study population, frequency of placenta accreta spectrum (PAS) and intraoperative blood loss (IOBL), and uterine artery (UtA) Doppler values in the complete group and in those with and without placenta previa.

	All (*n* = 177)	Previa (*n* = 129)	No previa (*n* = 48)	*p* value^*^
Age (years) median (range)	32 (22–43)	32 (22–43)	32 (23–40)	0.47
Body mass index median (range)	31 (20–53)	32 (20–53)	31 (23–53)	0.70
Gravidity median (range)	4 (1–8)	4 (2–8)	4 (1–8)	0.93
Parity median (range)	2 (0–5)	2 (0–5)	2 (0–5)	0.89
Number of prior cesarean deliveries median (range)	2 (0–5)	2 (0–5)	2 (0–5)	0.43
In vitro fertilization *n* (%)	6 (3.4)	3 (2.3)	3 (6.3)	0.20
History of myomectomy *n* (%)	5 (2.8)	2 (1.6)	4 (8.3)	**0.03**
Gestational age at referral (weeks) median (range)	28 (18–34)	27 (20–34)	29 (18–34)	0.09
No PAS *n* (%)	69 (39.0)	31 (24.0)	38 (79.2)	**<0.001**
PAS *n* (%)	108 (61.0)	98 (75.9)	10 (20.8)	**<0.001**
Placenta accreta *n* (%)	14/108 (12.9)	13/98 (13.2)	1/10 (10)	0.77
Placenta increta *n* (%)	66/108 (61.1)	61/98 (62.2)	5/10 (50)	0.45
Placenta percreta *n* (%)	28/108 (25.9)	24/98 (24.5)	4/10 (40)	0.29
Intraoperative blood loss (mL) median (range)	1500 (300–25,000)	1500 (500–15,000)	800 (300–25,000)	**<0.001**

* *p* value is for comparisons between patients with or without placenta previa.

Bold values indicate statistical significance *p* < 0.05.

### 3.1 UtA Doppler indices and PAS

**FIGURE 2:**
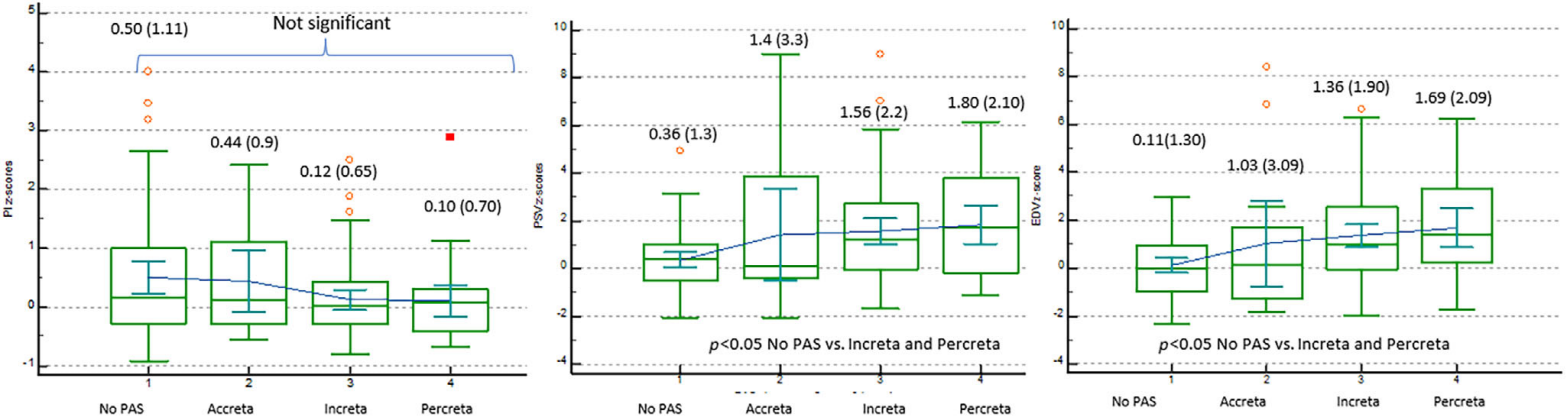
Uterine artery (UtA) Doppler indices stratified by depth of placental invasion. Doppler values were converted to gestational age–adjusted *z*‐scores. The mean *z*‐score and standard deviation are listed above each plot. Significant differences were observed in peak systolic (PSV) and end‐diastolic (EDV) velocities between patients with and without placenta increta or percreta.

Table 2 summarizes UtA Doppler *z*‐scores in the overall cohort and stratified by placenta previa status. In the complete cohort, patients with PAS had lower UtA‐PI and higher UtA‐PSV and UtA‐EDV compared with patients without PAS (all *p* < 0.05). Among patients with placenta previa, similar differences were observed. In contrast, among patients without placenta previa, those with PAS had significantly higher UtA‐PSV and UtA‐EDV, while UtA‐PI did not differ significantly. No significant differences in Doppler indices were observed across stages of PAS severity (accreta vs. increta vs. percreta) (Figure 2). One‐way ANOVA demonstrated significant differences between patients without PAS and those with increta or percreta, but not between no PAS and accreta, nor among PAS severity subgroups.

**TABLE 2:** Differences in uterine artery (UtA) Doppler parameters between cases presenting with or without placenta accreta spectrum (PAS), and between those presenting with or without severe intraoperative blood loss (IOBL), defined as >1500 mL. UtA Doppler values have been converted to gestational age–adjusted *z*‐scores. Values are reported as mean (standard deviation).

Placenta accreta spectrum									
	All (*n* = 177)			Placenta previa (*n* = 129)			No placenta previa (*n* = 48)		
	PAS	No PAS	*p* value	PAS	No PAS	*p* value	PAS	No PAS	*p* value
PI	0.15 (0.70)	0.50 (1.11)	**0.01**	0.17 (0.73)	0.55 (0.89)	**0.02**	0.01 (0.45)	0.46 (1.3)	0.3
PSV	1.61 (2.3)	0.36 (1.32)	**0.0001**	1.60 (2.3)	0.86 (1.6)	0.1	1.67 (2.8)	−0.01 (0.9)	**0.002**
EDV	1.40 (2.15)	0.11 (1.26)	**0.0001**	1.40 (2.1)	0.47 (1.5)	**0.03**	1.58 (2.7)	−0.16 (1.02)	**0.002**

Severe intraoperative blood loss (>1500 mL)									
	All			Placenta previa			No placenta previa		
	IOBL (+)	IOBL (−)	*p* value	IOBL (+)	IOBL (−)	*p*‐value	IOBL (+)	IOBL (−)	*p*‐value
PI	0.13 (0.70)	0.32 (0.80)	0.1	0.16 (0.8)	0.39 (0.70)	0.1	−0.01 (0.4)	0.23 (0.9)	0.4
PSV	1.69 (2.20)	0.54 (1.79)	**0.0004**	1.71 (2.1)	0.95 (2.0)	**0.05**	1.51 (2.7)	−0.10 (1.0)	**0.007**
EDV	1.50 (2.10)	0.35 (1.60)	**0.0001**	1.50 (1.90)	0.65 (1.80)	**0.02**	1.41 (2.6)	−0.13 (1.1)	**0.009**

Abbreviations: EDV, end‐diastolic velocity; PI, pulsatility index; PSV, peak systolic velocity.

Bold values indicate statistical significance *p* < 0.05.

### 3.2 UtA Doppler velocimetry and severe IOBL

Reliable IOBL estimates were available for 165 patients. Severe IOBL (>1500 mL) occurred in 86/165 (52.1%). Patients with severe IOBL had significantly higher UtA‐PSV and UtA‐EDV compared with those without severe IOBL (*n* = 79, 47.9%), while UtA‐PI did not differ significantly. Similar findings were observed when stratified by placenta previa status (Table 2).

### 3.3 Predictive performance for PAS and severe IOBL

Predictive performance metrics are shown in Table 3. In the overall cohort, optimal cut‐off values for PAS prediction were as follows: UtA‐PI ≤−0.71 *z*‐scores (25th percentile); UtA‐PSV ≥1.29 *z*‐scores (90th percentile); UtA‐EDV ≥1.29 *z*‐scores (95th percentile). Increased UtA velocities demonstrated sensitivities of 44.8%–49.5% and specificities of 81.2%–83.5% for PAS (Table 3). The predictive performance of UtA Doppler velocimetry for PAS seems to improve when velocities are analyzed primarily in those without placenta previa at risk of PAS (for UtA‐PSV ≥90 percentile for GA, sensitivity 60.0%, specificity 97.4%, PPV 85.8%, and NPV 90.3%; for UtA‐EDV ≥90 percentile, sensitivity 50.0%, specificity 94.7%, PPV 71.4%, and NPV 87.8%). Similar performance was observed for UtA‐PSV and UtA EDV for severe blood loss (Table 3). UtA‐PSV ≥1.29 *z*‐scores demonstrated 47.6% sensitivity and 85.9% specificity for prediction of severe blood loss.

**TABLE 3:** Performance of uterine artery (UtA) Doppler indices for the prediction of placenta accreta spectrum (PAS) and severe intraoperative blood loss (IOBL), defined as >1500 mL. UtA Doppler values have been converted to gestational age–adjusted *z*‐scores.

Placenta accreta spectrum									
	UtA‐PI			UtA‐PSV			UtA‐EDV		
	All	Previa	No previa	All	Previa	No previa	All	Previa	No previa
AUC‐ROC	0.57	0.64	0.52	0.65	0.57	0.77	0.68	0.62	0.75
Optimal cut‐off (z‐sc)	−0.71	−0.13	−0.89	1.29	2.50	1.29	1.29	2.70	1.29
Sens (%)	86.8	60.4	100	49.5	34.2	60.0	44.8	24.7	50.0
Spec (%)	31.3	65.4	26.3	83.5	89.6	97.4	81.2	96.5	94.7
PPV (%)	66.4	84.6	26.3	82.4	91.1	85.8	78.3	95.7	71.4
NPV (%)	60.3	34.4	100	51.4	30.2	90.3	49.1	28.9	87.8

Severe intraoperative blood loss (>1500 mL)									
	UtA‐PI			UtA‐PSV			UtA‐EDV		
	All	Previa	No previa	All	Previa	No previa	All	Previa	No previa
AUC‐ROC	0.58	0.63	0.51	0.66	0.61	0.74	0.68	0.64	0.73
Cut‐off (z‐sc)	0.01	0.04	0.30	1.87	1.87	1.13	0.81	1.78	1.50
Sens (%)	54.2	56.9	9.1	47.6	50.7	54.6	59.2	45.2	81.8
Spec (%)	60.3	68.7	70.0	85.9	77.1	86.7	70.5	81.3	63.3
PPV (%)	59.8	60.4	8.9	78.6	65.0	56.9	68.6	67.0	41.7
NPV (%)	54.8	65.5	70.6	60.1	65.1	85.6	61.4	63.9	91.6

Abbreviations: AUC‐ROC, area under the curve for the receiver operating characteristics curve; EDV, end‐diastolic velocity; NPV, negative predictive value; PI, pulsatility index; PPV, positive predictive value; PSV, peak systolic velocity; Sens, sensitivity; Spec, specificity; z‐sc, *z*‐scores.

### 3.4 Associations between UtA Doppler parameters, PAS, and severe IOBL

Logistic regression analyses are presented in Table 4. UtA‐PSV ≥1.29 *z*‐scores was significantly associated with PAS, OR 4.9 (95% CI, 2.31–10.39; *p* < 0.0001), and severe IOBL, OR 3.67 (95% CI, 1.86–7.23; *p* = 0.002). UtA‐EDV ≥1.29 *z*‐scores was significantly associated with PAS, OR 3.49 (95% CI, 1.71–7.14; *p* = 0.0006) and severe IOBL, OR 3.34 (95% CI, 1.68–6.64; *p* = 0.0006). Among patients without placenta previa, associations were markedly stronger. For PAS, UtA‐PSV ≥ 1.29 *z*‐scores was associated with PAS: OR 55.5 (95% CI, 5.2–584; *p* = 0.008), as was UtA‐EDV > 1.29 *z*‐scores: OR 18.0 (95% CI, 2.73–118.9; *p* = 0.003). For Severe IOBL, UtA‐PSV ≥ 1.29 *z*‐scores was associated with severe IOBL: OR 11.67 (95% CI, 1.81–75.1; *p* = 0.009), as was UtA‐EDV ≥1.29 *z*‐scores: OR 8.57 (95% CI, 1.30–56.53; *p* = 0.03).

**TABLE 4:** Logistic regression analyses between uterine artery (UtA) Doppler indices and their associations with placenta accreta spectrum (PAS), and severe intraoperative blood loss (IOBL), defined as >1500 mL.

Complete study group	OR (95% CI)	*p* value
Association with PAS		
PI ≤ −0.71 SD	0.71 (0.23–2.23)	0.2
PSV ≥ 1.29 SD	4.9 (2.31–10.39)	**<0.0001**
EDV ≥ 1.29 SD	3.49 (1.71–7.14)	**0.0006**
Association with severe IOBL		
PI ≤ −0.71 SD	1.34 (0.8–4.42)	0.6
PSV ≥ 1.29 SD	3.67 (1.86–7.23)	**0.002**
EDV ≥ 1.29 SD	3.34 (1.68–6.64)	**0.0006**
Patients without placenta previa		
Association with PAS		
PSV ≥ 1.29 SD	55.5 (5.2–584.0)	**0.008**
EDV ≥ 1.29 SD	18.0 (2.73–118.90)	**0.003**
Association with severe IOBL		
PSV ≥ 1.29 SD	11.67 (1.81–75.1)	**0.009**
EDV ≥ 1.29 SD	8.57 (1.30–56.53)	**0.03**

Abbreviations: CI, confidence intervals; EDV, end‐diastolic velocity; OR, odds ratio; PI, pulsatility index; PSV, peak systolic velocity; SD, standard deviation.

Bold values indicate statistical significance *p* < 0.05.

## 4 DISCUSSION

The principal findings of this study are as follows: (1) patients with PAS demonstrated significantly lower UtA‐PI and higher UtA‐PSV and UtA‐EDV compared with those without PAS; (2) UtA Doppler parameters did not change in relation to the depth of placental invasion; (3) the predictive performance of UtA Doppler velocimetry for PAS improved among patients without placenta previa; (4) those who experienced severe IOBL (>1500 mL) had significantly higher UtA‐PSV and UtA‐EDV compared with those without severe IOBL. These findings suggest that UtA Doppler evaluation may provide complementary hemodynamic information in the assessment of those at risk for PAS.

### 4.1 Our results in relation to what is known

Multiple prior studies have reported an increased risk of depth of placental implantation and greater blood loss at the time of delivery when increased vascularity is seen [27, 28, 29]. There is limited data characterizing UtA Doppler velocimetry in patients with PAS. Previous studies have reported reduced UtA PI and/or resistance index (RI) in patients with placenta previa complicated by PAS compared with placenta previa alone. Cho et al. reported decreased UtA‐PI in PAS and suggested that reduced uterine arterial resistance, combined with maternal risk factors (e.g., prior cesarean delivery, parity, abortion history, and maternal age), may improve prediction of placenta accreta [17]. Two prior studies evaluated UtA PSV. Vadhani et al. reported higher UtA‐PSV among patients with PAS who experienced severe hemorrhage requiring hysterectomy [19]. Zheng et al. described increased UtA‐PSV and decreased UtA‐PI/RI in PAS, reporting a high AUC (0.952) for UtA‐PSV in PAS prediction [20]. Our findings are consistent with prior reports demonstrating reduced PI and increased velocities in PAS, albeit with weaker associations. However, our study provides additional information: Doppler values were normalized for gestational age using *z*‐scores, EDV was also evaluated and showed a significant association with PAS, we constructed reference ranges of UtA velocities, and we analyzed the associations between UtA Doppler indices and severe IOBL.

To our knowledge, no prior studies have reported on the utility of UtA Doppler indices for the detection of PAS in patients without placenta previa. Interestingly, Doppler differences between PAS and non‐PAS cases were more pronounced in those without placenta previa (complete cohort: ROC‐AUC for UtA‐PSV and UtA‐EDV 0.65 and 0.68, respectively; no placenta previa: ROC‐AUC for UtA‐PSV and UtA EDV 0.77 and 0.75, respectively). However, this subgroup included relatively few PAS cases, and these findings should be interpreted cautiously. One possible explanation is that placenta previa itself may alter UtA hemodynamics, as patients with placenta previa, even without PAS, appear to exhibit lower PI and higher velocities compared to those without previa.

### 4.2 Clinical implications

The most common Doppler parameter evaluated from the UtAs is the PI. This index has been used in the identification of patients at risk of preeclampsia, fetal growth restriction, and as a potential marker for adverse perinatal outcomes. However, PI reflects downstream vascular resistance rather than true volumetric blood flow. In contrast, PSV and EDV represent more direct measures of blood flow and may better reflect the vascular changes associated with abnormal placentation. Two technical considerations are essential when evaluating UtA velocities: (1) the angle of insonation should be maintained as close to 0° as possible, optimizing probe orientation and applying angle correction when necessary, and (2) the Doppler sample volume should be placed approximately 1 cm from the crossing with the external iliac artery to ensure reproducibility [30].

Evaluation of the UtA seems to be more useful in patients with no placenta previa; UtA velocities for the prediction of PAS in this subgroup (when ≥90th percentile for GA) showed a sensitivity of 50.0%–60.0% and a specificity of 94.7%–97.7%. More commonly used vascular ultrasound markers for the prediction of PAS include placental lacunae, increased uterovesical hypervascularity, and increased subplacental vascularity with sensitivity ranging from 51.3% to 78.5% and specificity ranging from 80.9% to 99.4% for these markers, though this is primarily validated in patients with placenta previa [31, 32]. Cases of PAS in the absence of placenta previa are rarer, and transvaginal ultrasound provides limited information. The UtA Doppler evaluation can contribute to the suspicion of PAS in patients with risk factors at a higher risk of PAS but without placenta previa (e.g., IVF pregnancies, previous uterine curettage, myomectomy, or classical cesarean incision).

When UtA‐PSV or UtA‐EDV are elevated, this can increase suspicion of the presence of PAS. Nevertheless, the individual clinical contribution of the UtA seems to be limited in the absence of other signs of PAS. The individual contribution of the UtA as compared to other known signs of PAS should be evaluated in a larger sample size.

### 4.3 Strengths and limitations

Strengths of this study include the prospective design, standardized ultrasound assessment at a single quaternary center, and histopathologic confirmation of PAS. This is one of the first studies to systematically evaluate UtA‐PSV and UtA‐EDV in addition to PI in this population. UtA Doppler is widely available, relatively simple to perform, and offers a quantitative assessment of uteroplacental hemodynamics.

Our study is not without limitations. First, the number of PAS cases without placenta previa was small, limiting statistical precision in subgroup analyses. Second, patients managed conservatively were excluded, as histopathologic confirmation was not available; this may limit generalizability. Third, *z*‐score normalization relied on general reference values rather than reference ranges specific to placental location. The proportion of severe PAS (increta/percreta) was higher than typically reported, likely reflecting referral bias to a quaternary center and/or differences in histopathologic classification. Finally, our patients were referred to our center because there was concern for PAS from the referring clinician, indicating that our study population had a high a priori risk and limiting generalizability of our findings for low‐risk patients.

## 5 CONCLUSION

Patients with PAS exhibit lower UtA‐PI and higher UtA‐PSV and UtA‐EDV compared to those without PAS, and when UtA‐PSV or UtA‐EDV are above the 90%ile for gestational age, this can increase suspicion for the presence of PAS. The predictive performance of UtA Doppler velocimetry appears stronger in the absence of placenta previa. Elevated UtA velocities are associated with severe IOBL and may assist in perioperative risk stratification. Further studies are needed to define the value of UtA Doppler indices relative to established sonographic markers of PAS.

## CONFLICT OF INTEREST STATEMENT

The authors declare no conflicts of interest.

## FUNDING INFORMATION

The authors received no specific funding for this work.

## ETHICS STATEMENT

This study was approved by the institutional review board from the University of Texas Health Science Center at Houston (HSC‐MS‐18‐0968). Data evaluation complies with the guidelines for human studies, and data collection was conducted ethically in accordance with the World Medical Association and the Declaration of Helsinki. All data generated or analyzed during this study are included in this article. Further inquiries can be directed to the corresponding author.

## Supporting information



Supporting file 1: pmf270436‐sup‐0001‐SuppMat.docx

## Data Availability

The data that support the findings of this study are not publicly available due to privacy/ethical restrictions but are available from the corresponding author upon reasonable request.
