# Supplementary material for: Association between uterine artery Doppler velocimetry, placenta accreta spectrum, and depth of placental implantation
Source: Pregnancy (Hoboken). 2026 Aug 27;2(5):e70436. doi: 10.1002/pmf2.70436 (PMC13520923; doi:10.1002/pmf2.70436)
Supplement: Supplementary file 1 [file PMF2-2-e70436-s001.docx]

**Supplemental Table 1.** Reference values for uterine artery pulsatility index (PI), peak systolic velocity (PSV [cm/s]) and end diastolic velocity (EDV [cm/s])

| Weeks | PI 10th | PI 50th | PI 90th |  | PSV 10th | PSV 50th | PSV 90th |  | EDV 10th | EDV 50th | EDV 90th |
| --- | --- | --- | --- | --- | --- | --- | --- | --- | --- | --- | --- |
| 20.0 | 0.51 | 0.98 | 1.45 |  | 53.62 | 93.55 | 133.48 |  | 20.31 | 41.71 | 63.11 |
| 21.0 | 0.50 | 0.94 | 1.38 |  | 55.05 | 94.82 | 134.59 |  | 21.96 | 43.28 | 64.60 |
| 22.0 | 0.49 | 0.91 | 1.32 |  | 56.41 | 96.09 | 135.77 |  | 23.49 | 44.79 | 66.09 |
| 23.0 | 0.48 | 0.88 | 1.27 |  | 57.70 | 97.36 | 137.02 |  | 24.89 | 46.25 | 67.61 |
| 24.0 | 0.47 | 0.85 | 1.22 |  | 58.91 | 98.63 | 138.35 |  | 26.17 | 47.66 | 69.15 |
| 25.0 | 0.46 | 0.82 | 1.18 |  | 60.04 | 99.90 | 139.76 |  | 27.31 | 49.01 | 70.70 |
| 26.0 | 0.45 | 0.80 | 1.15 |  | 61.10 | 101.17 | 141.24 |  | 28.34 | 50.30 | 72.27 |
| 27.0 | 0.44 | 0.78 | 1.12 |  | 62.09 | 102.44 | 142.79 |  | 29.23 | 51.54 | 73.85 |
| 28.0 | 0.43 | 0.76 | 1.10 |  | 62.99 | 103.71 | 144.43 |  | 30.00 | 52.73 | 75.46 |
| 29.0 | 0.41 | 0.75 | 1.09 |  | 63.83 | 104.98 | 146.13 |  | 30.64 | 53.86 | 77.08 |
| 30.0 | 0.40 | 0.74 | 1.08 |  | 64.58 | 106.25 | 147.92 |  | 31.15 | 54.94 | 78.72 |
| 31.0 | 0.38 | 0.73 | 1.08 |  | 65.26 | 107.52 | 149.78 |  | 31.54 | 55.96 | 80.38 |
| 32.0 | 0.37 | 0.73 | 1.09 |  | 65.87 | 108.79 | 151.71 |  | 31.80 | 56.93 | 82.05 |
| 33.0 | 0.35 | 0.73 | 1.10 |  | 66.40 | 110.06 | 153.72 |  | 31.93 | 57.84 | 83.75 |
| 34.0 | 0.34 | 0.73 | 1.12 |  | 66.86 | 111.33 | 155.80 |  | 31.94 | 58.70 | 85.46 |
| 35.0 | 0.32 | 0.73 | 1.15 |  | 67.23 | 112.60 | 157.96 |  | 31.81 | 59.50 | 87.19 |
| 36.0 | 0.30 | 0.74 | 1.18 |  | 67.54 | 113.87 | 160.20 |  | 31.57 | 60.25 | 88.93 |
| 37.0 | 0.29 | 0.75 | 1.22 |  | 67.77 | 115.14 | 162.51 |  | 31.19 | 60.94 | 90.69 |
| 38.0 | 0.27 | 0.77 | 1.26 |  | 67.92 | 116.41 | 164.90 |  | 30.69 | 61.58 | 92.48 |
| 39.0 | 0.25 | 0.78 | 1.32 |  | 68.00 | 117.68 | 167.36 |  | 30.06 | 62.17 | 94.27 |
| 40.0 | 0.23 | 0.80 | 1.38 |  | 68.00 | 118.95 | 169.90 |  | 29.31 | 62.70 | 96.09 |
| 41.0 | 0.21 | 0.82 | 1.44 |  | 67.92 | 120.22 | 172.51 |  | 28.43 | 63.18 | 97.92 |
